# Development and validation of serological dynamic risk score to predict outcome in gastric cancer with adjuvant chemotherapy: a multicentre, longitudinal, cohort study

**DOI:** 10.3389/fonc.2024.1327691

**Published:** 2024-02-20

**Authors:** Linbin Lu, Wenzheng Fang, Jun Yu, Xianchun Gao, Xinlin Wang, Yan Pan, Weili Han, Junya Yan, Huahong Xie, Liping Yao, Jianjun Yang, Jianyong Zheng, Liu Hong, Jipeng Li, Mengbin Li, Lei Shang, Kaichun Wu, Gang Ji, Yongzhan Nie

**Affiliations:** ^1^ State Key Laboratory of Holistic Integrative Management of Gastrointestinal Cancers and National Clinical Research Center for Digestive Diseases, Xijing Hospital of Digestive Diseases, Fourth Military Medical University, Xi’an, China; ^2^ Department of Oncology, People’s Hospital Affiliated to Fujian University of Traditional Chinese Medicine, Fuzhou, China; ^3^ Department of Oncology, the 900th Hospital of Joint Logistic Support Force, Chinese People's Liberation Army (PLA), Fuzong Clinical College of Fujian Medical University, Fuzhou, Fujian, China; ^4^ Department of Medical Statistics, School of Preventive Medicine, Fourth Military Medical University, Xi’an, China

**Keywords:** gastric cancer, gastrectomy, adjuvant chemotherapy, predictive model, risk score, risk state chains

## Abstract

**Background:**

Baseline serological biomarkers have the potential to predict the benefits of adjuvant chemotherapy in patients with gastric cancer. However, the fluctuating nature of postoperative recurrence risk makes precise treatment challenging. We aimed to develop a risk score in real-time predicting outcomes for postoperative GC patients using blood chemistry tests.

**Materials and methods:**

This was a retrospective, multicentre, longitudinal cohort study from three cancer centres in China, with a total of 2737 GC patients in the pTNM stage Ib to III. Among them, 1651 patients with at least two serological records were assigned to the training cohort. Model validation was carried out using separate testing data with area under curve (AUC). The least absolute shrinkage and selection operator (LASSO) and random forest-recursive feature elimination (RF-RFE) algorithm were used to select the parameters.

**Results:**

The Cox regression model derived six risk factors to construct a composite score (low-risk: 0-2 score; high risk: 3-6 score), including CEA, CA125, CA199, haemoglobin, albumin, and neutrophil to lymphocyte ratio. The risk score accurately predicted mortality in 1000-time bootstrap (AUROCs:0.658; 95% CI: 0.645, 0.670), with the highest AUROC (0.767; 95% CI: 0.743, 0.791) after 1 year since the gastrectomy. In validation dataset, the risk score had an AUROC of 0.586 (95% CI 0.544, 0.628). Furthermore, patients with high risk at 1 month derived significant clinical benefits from adjuvant chemotherapy (*P* for interaction <0.0001). Compared with the low-low-low risk group, the low-low-high risk group of the long-term state chain (risk state at baseline, 6 months, 1 year) had the worse OS (HR, 6.91; 95%CI: 4.27, 11.19) and DFS (HR, 7.27; 95%CI: 4.55, 11.63).

**Conclusion:**

The dynamic risk score is an accurate and user-friendly serological risk assessment tool for predicting outcomes and assisting clinical decisions after gastrectomy.

## 1 Background

Gastric cancer (GC) is the third most prevalent cancer and the second leading cause of cancer-related deaths in China (1). It is the third leading cause of cancer-related deaths worldwide (2). Radical gastrectomy is the most effective treatment for resectable GC. However, within 5 years after curative resection with systemic therapies, approximately 6%–86% of patients with stage Ib to III GC will experience recurrence, which significantly contributes to death from GC (3). Tumour markers, such as CEA and CA19-9, are extensively utilised in clinical practice to effectively identify tumour recurrence, owing to their cost-effectiveness and ability to provide real-time monitoring. Several treatment guidelines advocate the early detection of tumour markers, which often precedes the detection of recurrence/metastasis by imaging examinations for 2–3 months (4–6).

A nationwide prospective observational study suggested that monitoring CEA and/or CA19-9 levels after surgery could help predict the recurrence of GC, especially in patients with high preoperative levels of these markers (6). Despite achieving a sensitivity of 85.0% for recurrence (4), the positive rates were 21.1% for CEA and 27.8% for CA19-9 (7). The combined use of CEA and CA19-9 has limitations regarding personalised prediction, and there is a pressing need to incorporate other serological tests. The Chinese and European treatment guidelines not only recommend these blood chemistries but also advocate for whole blood count and liver-renal function tests in China (4, 8). In addition, a previous study summarised several immunological and nutritional factors that influence recurrence after radical surgery (9). These factors are associated with the clinical outcomes of patients with GC, including the neutrophil-to-lymphocyte ratio (NLR) (10, 11), lymphocyte–monocyte ratio (12) (LMR), platelet–lymphocyte ratio (PLR) (13, 14), and systemic immune-inflammation index (SII) (15). Factors related to the nutritional status of patients, such as serum albumin (16) and body mass index (17), also play a role. Furthermore, changes in perioperative LMR at different time points have helped predict the long-term survival of GC patients (18).

However, whether serial analysis of blood chemistry tests offer real-time insights into the effectiveness of adjuvant chemotherapy in patients with GC remains unclear. There is still no evidence of a comprehensive evaluation of the monitoring value of blood tumour markers and immuno-nutritional indices after surgery. In this study, we collected and analysed longitudinal blood chemistry data, including whole blood counts, liver function tests, and tumour marker levels, from a multicentre cohort. Our findings indicate that a composite score based on selected serological parameters can dynamically and accurately predict the risk of death after surgery. Additionally, we examined the predictive performance and survival of the short- and long-term risk state chains using composite scores.

## 2 Materials and methods

### 2.1 Study design and follow-up

This was a retrospective, multicentre, longitudinal cohort study from three cancer centres in China, with 2,737 patients with GC in TNM stages Ib to III. Among these patients, 1,651 with GC confirmed by at least two serological tests and admitted to the Xijing Hospital in Shanxi Province, China, from 1 January 2011 to 13 December 2016, were evaluated for inclusion. The final follow-up was on 5 November 2022. Additionally, out of the 2,737 patients, 1,086 with only baseline serological tests were enrolled from two hospitals in Fujian Province, China, from 1 January 2009 to 31 December 2011. Patients receiving preoperative neoadjuvant therapy were excluded for further analysis.

Most patients received adjuvant chemotherapy postoperatively. They were followed up every 3–6 months during the first 2 years and subsequently every 6–12 months up to year 5. After 5 years, follow-ups were conducted annually.

This study was approved by the Ethics Committee of the Hospital (Approval No. KY20192088-F-1). Due to the retrospective design of this study, informed consent was not required.

### 2.2 Data collection and definitions

We gathered demographic information, clinical or pathological characteristics, laboratory test results, and outcome data of the training cohort from the database of Xijing Hospital. Data from two Fujian hospitals were obtained as validation cohorts using data collection forms from patients’ electronic medical records.

Serological values were extracted, including complete blood counts (WBC count, neutrophil count, lymphocyte count, monocyte count, haemoglobin concentration, and platelet count), tumour biomarkers (CEA, CA199, CA125, and AFP), and serum albumin. Preoperative serological values were defined as those closest to gastrectomy without any treatment. Postoperative variables were recorded in each follow-up before any treatment. Besides, only the first records were included for patients with repeated serological tests at each follow-up visit. The neutrophil/lymphocyte ratio (NLR), platelet/lymphocyte ratio (PLR), lymphocyte/monocyte ratio (LMR), and systemic immune inflammation index (SII, platelet×neutrophil/lymphocyte) were calculated and used as candidate predictors because of their significant prognostic value for GC.

The primary outcome was overall survival (OS), from surgery to all-cause mortality. Additionally, disease-free survival is considered a secondary outcome from surgery to recurrence or all-cause mortality.

### 2.3 Predictor selection and model development

**Figure 1:**
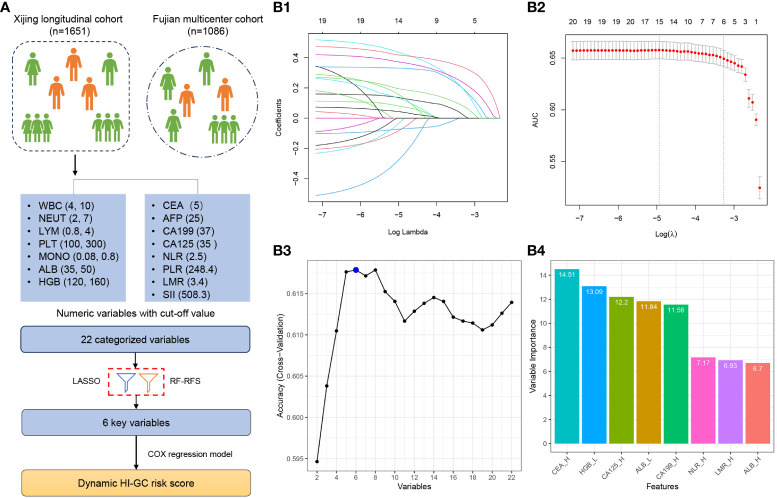
Study and Variable Selection Flowchart. **(A)** A total of 22 categorized variables were initially considered for inclusion in the selection process using the Xijing longitudinal cohort. Eventually, 6 variables were chosen to develop the risk assessment score. **(B1)** Cross-validation plot for determining the penalty term, and **(B2)** plots showing the regression coefficients obtained through LASSO regression for different penalty parameter values. **(B3)** The number of features with optimal accuracy under 10-fold cross-validation in FR-RFE, and **(B4)** Variable importance in FR-RFE. LASSO, Least Absolute Shrinkage and Selector Operation; RF-RFE, Random Forest Recursive Feature Elimination.

**Figure 1** shows the variable selection process. All patients in the training cohort were included in the variable selection and model development. To improve the risk score, 7 of the 15 variables (complete blood counts and serum albumin) were transformed into two categorical variables representing > upper or < lower limit of normal. Additionally, four tumour biomarkers were transformed into CEA (CEA_H, > 5 ng/mL), CA199 (CA199_H, > 37 U/mL), CA125 (CA125_H, > 35 U/mL), and AFP (AFP_H, > 25 ng/mL) increase according to the upper limit of normal. Based on the recent studies, NLR, PLR, LMR, and SII were transformed into two categorical variables (NLR_H, > 2.5 (10, 14); PLR_H, > 248.4 (14); LMR_H, > 3.4 (18); SII_H, > 508.3 (19)). These cut-off values were further validated in the training cohort at baseline through the receiver operating characteristic (ROC) curve (**Supplementary Table S1**). Thus, 22 variables were used for subsequent variable selection.

Subsequently, the least absolute shrinkage and selection operator (LASSO) algorithm was implemented to select potential predictive factors using penalty parameter tuning through 10-fold cross-validation. Additionally, the random forest-recursive feature elimination (RF-RFE) algorithm was employed in parallel for factor selection. Finally, we merged the factors selected by the LASSO and RF-RFE algorithms.

### 2.4 Score development and validation

To validate the predictive ability of the fixed effects and enhance clinical usability, we assigned a numerical score to each of the final selected predictors proportional to its specific coefficient in the Cox proportional hazards regression model. This allowed us to develop a composite score and calculate the dynamic risk scores for each patient based on their serological records over 3 years. To assess the model’s overall performance in predicting the risk of death, we conducted a covariate-specific time-dependent ROC curve analysis in the training cohort. The HI-GC risk score derived from the Cox model was validated externally.

To evaluate the prediction accuracy across different follow-up periods, we divided the follow-up periods into quartiles. We assessed the average area under the receiver operating characteristic (AUROC), sensitivity, and specificity of the mean score within each quartile. Based on clinical practice and 8,395 observations, we identified four optimal subgroups for further analysis: Q1 (0–1 month), Q2 (1–6 months), Q3 (6–12 months), and Q4 (> 12 months). Subsequently, we further validated the score’s performance in 1,086 patients from the Fujian Province using only baseline variables.

### 2.5 Risk state transition analysis

Survival was calculated using the Kaplan–Meier method with a log-rank test, and univariate comparisons were performed using an unadjusted Cox model. Missing longitudinal data were handled using multiple imputations (MI) with a random forest. A linear fitting curve for the dynamic serological parameter trajectories was plotted using a generalised additive model.

The HI-GC score categorises patients into the low- (HI-GC <3) and high-risk (HI-GC ≥3) groups based on the 80th percentile. To analyse the longitudinal risk state transitions further, we established short- and long-term state chain models: (1) risk state at baseline and 1 month after surgery, and (2) risk state at baseline, 6 months, and 12 months. The risk state at 1 month was defined as the first HI-GC score for Q2 (1–6 months). Similarly, the first scores for Q3 (6–12 months) and Q4 (> 12 months) were for 6 and 12 months, respectively. We used this model to estimate the mean score of each subgroup and the proportion of transitions between subgroups at subsequent time points simultaneously. Harrell’s C-index was used as a discrimination measure for short- and long-term state chains.

All statistical analyses were performed using R-3.6.3. Statistical significance was defined by a two-sided P value less than 0.05.

## 3 Results

### 3.1 Clinical characteristics of patients

A total of 1,651 patients with 8,395 hospitalisation or follow-up records were included in the analysis of the training cohort. The baseline clinical characteristics are shown in **Table 1**. The participants’ median age was 56.9 years (SD, 10.6), and 77.3% were males. Gastrectomy was used as frontline treatment in patients with AJCC 8th TNM stages Ib (n=140, 8.5%), II (n=485, 29.4%), and III (n=1026, 62.1%). The majority (n=1,468, 88.9%) were treated with adjuvant chemotherapy after surgery. The median follow-up time was 5.8 (IQR, 2.0–7.9) years. All patients underwent radical resection of gastric cancer with negative surgical margins.

**Table 1:** Baseline characteristics of the training cohort group by HI-GC risk score.

	Risk score		P-value
	Low	High	
N	1264	387	
Age	56.5 ± 10.5	58.3 ± 10.6	0.003
BMI	22.5 ± 2.9	21.9 ± 2.8	<0.001
Sex			0.667
Male	980 (77.5%)	296 (76.5%)	
Female	284 (22.5%)	91 (23.5%)	
Type of gastrectomy			0.386
Proximal	102 (8.1%)	24 (6.2%)	
Distal	519 (41.1%)	155 (40.1%)	
Total	643 (50.9%)	208 (53.7%)	
Differentiation			0.003
Well	127 (10.0%)	52 (13.4%)	
Moderate	176 (13.9%)	74 (19.1%)	
Poor/Undifferentiation	961 (76.0%)	261 (67.4%)	
Primary tumour location			0.612
Proximal	407 (32.2%)	122 (31.5%)	
Body	368 (29.1%)	105 (27.1%)	
Antrum	489 (38.7%)	160 (41.3%)	
Lymphovascular invasion			0.110
No	397 (31.4%)	105 (27.1%)	
Yes	867 (68.6%)	282 (72.9%)	
Nerve invasion			0.048
No	108 (8.5%)	46 (11.9%)	
Yes	1156 (91.5%)	341 (88.1%)	
pN stage			<0.001
0	346 (27.4%)	64 (16.5%)	
1	249 (19.7%)	71 (18.3%)	
2	276 (21.8%)	81 (20.9%)	
3	393 (31.1%)	171 (44.2%)	
pT stage			<0.001
1	2 (0.2%)	0 (0.0%)	
2	262 (20.7%)	39 (10.1%)	
3	345 (27.3%)	111 (28.7%)	
4	655 (51.8%)	237 (61.2%)	
pTNM stage			<0.001
I	126 (10.0%)	14 (3.6%)	
II	399 (31.6%)	86 (22.2%)	
III	739 (58.5%)	287 (74.2%)	

Mean±SD/N (%). Differences are compared using the chi-square test (or Fisher's exact test) for categorical measures and Kruskal−Wallis test for continuous measures.

**Supplementary Figure S1** illustrates the linear fitting curve for the dynamic trajectories of the 15 serological parameters from surgery to 3 years of follow-up. The ALB and HGB levels increased over time after surgery, whereas CEA, CA199, CA125, and NLR decreased steadily during the first 2 weeks, followed by a smooth trend in the later period.

### 3.2 Predictor selection and score development

All 1,651 patients with 8,395 observation data points in the training cohort were included for variable selection and risk score development. The variable selection process is illustrated in **Figure 1A**. After discretisation, the 22 categorised variables were used for subsequent variable selection.

Next, we used two different machine learning algorithms to select the most significant variable for classifying the overall survival of patients. First, a set of six variables (CEA_H, HGB_L, CA125_H, ALB_L, CA199_H, and NLR_H) was identified using the LASSO algorithm (**Figures 1B1, B2**). Second, we used the RF-RFE algorithm and selected the same set of six variables (**Figure 1B3**). We found that CEA_H, HGB_L, CA125_H, ALB_L, CA199_H, and NLR_H were the six most important variables in predicting the risk of death (**Figure 1B4**).

We then assigned each factor a numeric score by rounding the number of specific coefficients in the Cox proportional hazards regression model. Thus, a risk assessment scoring model (HI-GC risk score) was established based on six predictors (CEA_H, HGB_L, CA125_H, ALB_L, CA199_H, and NLR_H), with possible scores ranging from 0 to 6 (**Table 2**).

**Table 2:** Point distribution according to the coefficiency in the Cox model for the longitudinal cohort.

Covariates	β	SE	exp(β)	*P-*value	Points
CEA increase	0.32	0.04	1.38	<0.0001	1
CA125 increase	0.15	0.05	1.17	0.0014	1
CA199 increase	0.28	0.04	1.32	<0.0001	1
ALB decrease	0.26	0.05	1.30	<0.0001	1
HGB decrease	0.32	0.03	1.38	<0.0001	1
NLR >2.5	0.23	0.03	1.26	<0.0001	1

CEA increase: CEA > 5ng/mL; CA125 increase: CA125 >35 U/mL; CA199 increase: CA199 > 37U/mL; ALB <35 g/L; HGB decrease < 120 g/L. NLR, neutrophil: lymphocyte ratio; SE, standard error.

Cox model: ℎ(t, X) = ℎ_0 (t) exp(0.32[CEA increase] + 0.15[CA125 increase] + 0.28[CA199 increase] + 0.26[ALB decrease] + 0.32[HGB decrease] + 0.23[NLR > 2.5]).

The frequency distribution chart of the HI-GC risk score is shown in **Supplementary Figure S2A**. Because the number of scores 0–2 accounts for 76.4% of all observational data, we set risk score 2 as the cutoff value. The patients were divided into the low- (scores 0–2) and high-risk groups (scores 3–6). **Supplementary Figure S2B** shows the mean trajectories of the HI-GC risk score stratified by 1-year disease-free survival during the 3-year follow-up period. In the disease progression group, the risk score decreased from an elevated preoperative level (> 3 points) to 2 points within 3 months of surgery and then increased. However, in the disease-free group, the risk score declined rapidly to 1 point within 3 months and remained stable.

### 3.3 Performance of HI-GC risk score in the training and validation cohorts

We performed internal validation of the accuracy of the HI-GC risk score using a 1,000-time bootstrap among patients with outcomes. The average AUROC curve for the 1,000 subsets from the training cohort was 0.658 (95% CI: 0.645, 0.670). On dividing the follow-up period into quartiles, we observed an increasing accuracy of the HI-GC risk score in predicting mortality across different time intervals. The lowest AUROC, 0.606 (95% CI: 0.583, 0.629), was observed at 0–1 month after surgery, while the highest AUROC, 0.767 (95% CI: 0.743, 0.791), was achieved at 12 months after surgery (**Supplementary Table S2**). Similar results were observed in additional subgroups, including patients aged <50 and ≥50 years, sex (female and male), TNM stages (I, II, and III), and primary tumour locations (proximal, body, and antrum) (**Supplementary Table S3**). The HI-GC risk score remained consistent across different patient subgroups. Additionally, among patients aged <50, the HI-GC risk score demonstrated the highest accuracy after 12 months (AUROC: 0.792; 95% CI: 0.746, 0.837). Similarly, for TNM stage I, the HI-GC risk score exhibited the highest accuracy (AUROC: 0.824; 95% CI: 0.560, 1.000).

**Supplementary Figure S3** showed the Kaplan–Meier survival curves according to the HI-GC risk score. At baseline, the median OS of the low-risk group was 11.43 years (95% CI: 9.44, NA), which was significantly higher than the high-risk group (3.32; 95% CI: 2.82, 4.58; P<0.0001) (**Supplementary Figure S3A1**). In addition, the median DFS of the low-risk group (9.44; 95% CI: 8.28, NA) was also greater than the high-risk group (2.57; 95% CI: 1.98, 3.73; P<0.0001) (**Supplementary Figure S3A2**). At 12 months, the median OS (2.09; 95% CI: 1.93, 2.50) and DFS (1.49; 95% CI: 1.37, 1.69) of the high-risk group were markedly lower than the low-risk group (median OS and DFS not reached; P<0.0001) (**Supplementary Figures S3B1, B2**). Compared with the low-risk group, hazard ratios (HRs) of OS and DFS for the high-risk group were 5.42 (95% CI: 4.17, 7.03) and 5.38 (95% CI: 4.15, 6.96), respectively. While at the baseline, HRs of OS and DFS for the high-risk group were 1.64 (95% CI: 1.41, 1.91) and 1.64 (95% CI: 1.42, 1.91), respectively.

In the validation dataset, the baseline clinical characteristics of the 1,086 patients from Fujian Province with only a single serological test were described in **Supplementary Table S4**. In this cohort, the HI-GC risk score had an AUROC of 0.586 (95% CI: 0.544, 0.628), a sensitivity of 0.485, a specificity of 0.647, and an accuracy of 0.623. At baseline, the HR of OS for the high-risk group was 2.19 (95% CI:1.39, 3.47; P<0.0006) when compared with the low-risk group (**Supplementary Figure S4**).

### 3.4 Performance and survival analysis of risk state transition chain in the training cohorts

**Figure 2:**
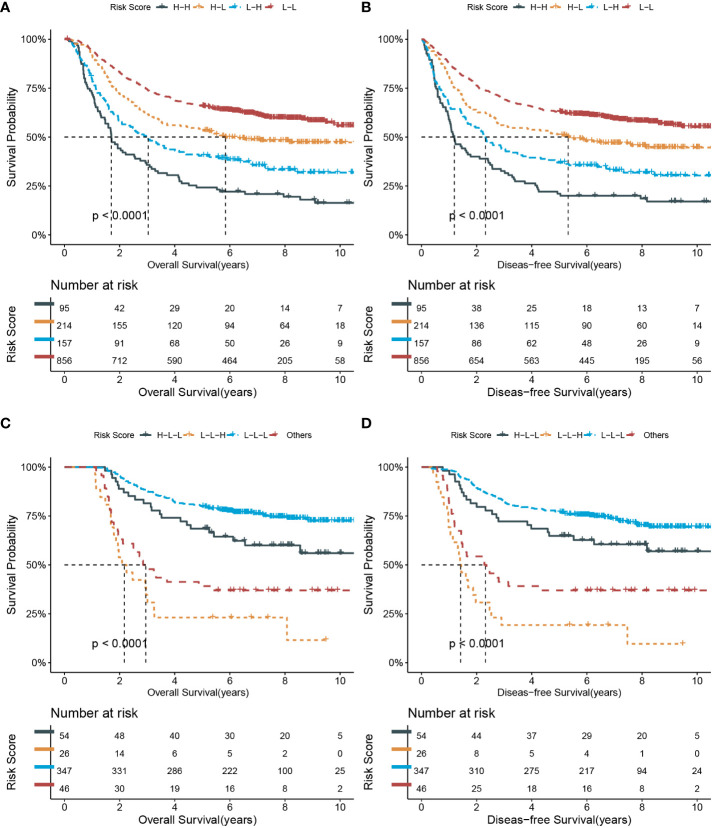
Kaplan-Meier survival curves of overall and disease-free survival according to short-term and long-term state chain models. **(A, B)** short-term risk state at baseline and 1 month; **(C, D)** long-term risk state at baseline, 6 and 12 months. L, low risk; H, high risk.

In the short-term state chain models, 1322 patients were selected with two test records at baseline and 1 month. Four state chains were identified: high-high risk (n=95, 7.2%), high-low risk (n=214, 16.2%), low-high risk (n=157, 11.9%), and low-low risk (n=856, 64.7%). The OS and DFS differed significantly among the four state chains, as shown in **Figures 2A, B** (All P <0.0001). Compared with the low-low risk group, the HRs of OS and DFS were 3.48 (2.72, 4.46) and 3.27 (2.56, 4.18) for the high-high risk group, respectively. Harrell’s C-index of the short-term state chain for OS was 0.603 (95% CI: 0.583, 0.623).

To explore whether the short-term state-chain model has predictive value for adjuvant chemotherapy. We performed a subgroup analysis to calculate the HRs of clinical outcomes for adjuvant chemotherapy stratified by state chain. As shown in **Table 3**, only patients in the high- and low-risk groups showed a significant clinical benefit from adjuvant chemotherapy (P <0.0001 for the interaction). HRs of adjuvant chemotherapy in the low-high risk group were 0.38 (95%CI: 0.20, 0.72) for OS and 0.44 (95%CI: 0.23, 0.82) for DFS. Besides, the HRs were 0.41 (95%CI: 0.21, 0.79) and 0.51 (95%CI: 0.26, 0.97) for OS and DFS in the high-high risk group, respectively. After adjustment for the PS score, patients in the high- and low-risk groups still had a significant clinical benefit from adjuvant chemotherapy (P <0.0001 for interaction).

**Table 3:** Hazard ratio (95% confidence intervals) of clinical outcome for adjuvant chemotherapy stratified by short-term state chain.

State chain	Event	N	Overall Survival		*P* value ^*^
			Unadjusted	Adjusted with PS score^#^	<0.0001
Low-Low	339	856	1.09 (0.74, 1.61)	0.80 (0.54, 1.19)	
High-Low	111	214	1.34 (0.59, 3.05)	1.06 (0.46, 2.47)	
Low-High	102	157	0.38 (0.20, 0.72)	0.29 (0.15, 0.57)	
High-High	78	95	0.41 (0.21, 0.79)	0.38 (0.20, 0.73)	
State chain	Event	N	Disease-free Survival		*P* value
			Unadjusted	Adjusted with PS score^#^	<0.0001
Low-Low	355	856	1.09 (0.75, 1.58)	0.83 (0.57, 1.21)	
High-Low	116	214	1.00 (0.49, 2.06)	0.74 (0.35, 1.57)	
Low-High	106	157	0.44 (0.23, 0.82)	0.34 (0.17, 0.67)	
High-High	78	95	0.51 (0.26, 0.97)	0.47 (0.24, 0.91)	

# PS score was calculated by age, sex, TNM stage, location, differential, lymphovascular invasion, and nerve invasion.

* All P values for interaction < 0.0001 before or after adjustment with PS score.

Low/High: Low risk or high risk at baseline or 1 month after surgery.

**Figure 3:**
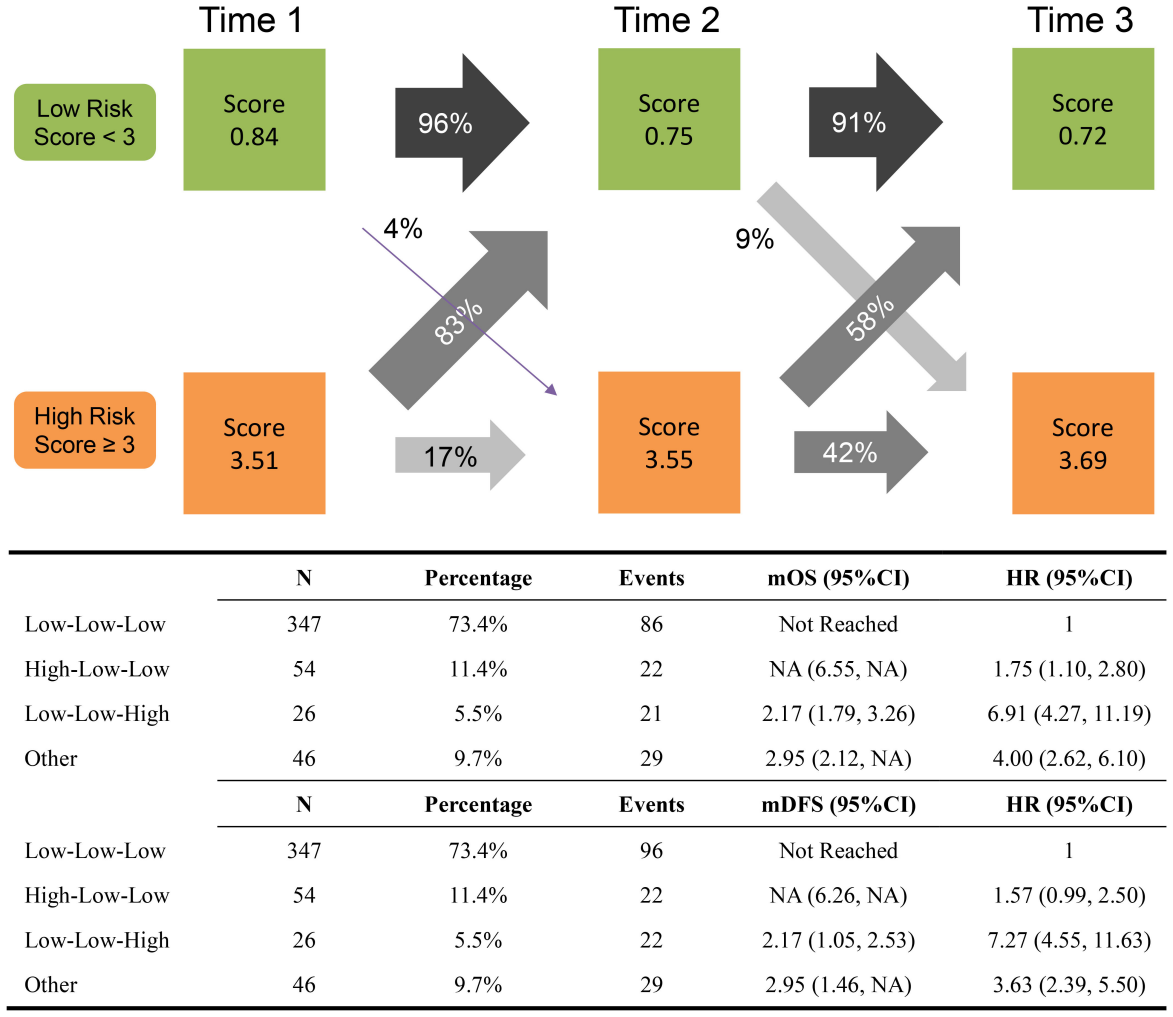
Long-term state chain prevalence and transition proportions between the states at subsequent time points. The mean HI-GC risk score of each status at each time point is recorded in the boxes. Time 1, time 2, and time 3 equals the time points of baseline, 6 months, and 12 months after surgery.

In total, 473 patients with medical records at baseline, 6 months, and 12 months were included in the long-term state-chain model. **Figure 3** shows the mean HI-GC risk score for the low-risk or high-risk group at the three-time points and the proportion of each risk state transitioning to the next time point. The top three state chains were low-low-low risk (L-L-L; n=347, 73.4%), high-low-low risk (H-L-L; n=54, 11.4%), and low-low-high risk (L-L-H; n=26, 5.5%). Moreover, the rest of the state chains were included in the other group (n= 46, 9.7%) because their percentages were below 5%. Of note, compared with the low-low-low risk group, the low-low-high risk group has the worse OS (HR, 6.91; 95%CI: 4.27, 11.19) and DFS (HR, 7.27; 95%CI: 4.55, 11.63) (also see **Figures 2C, D**). Harrell’s C-index of the long-term state chain for OS was 0.640 (95%: 0.622, 0.658).

## 4 Discussion

Serum biomarkers, such as tumour biomarkers, whole blood count, and liver renal function tests, are widely used in clinical settings to dynamically detect tumour recurrence, assess nutritional status, and evaluate potential chemotherapy toxicity. Although imaging studies are necessary to diagnose tumour recurrence, their high cost and limited applicability to larger tumours restrict their use for early detection. Numerous studies have demonstrated the prognostic and monitoring significance of CEA and/or CA199 (6, 20–24). The sensitivity of CEA or CA19-9 was higher in patients with elevated preoperative levels than in those with normal levels (25). Nevertheless, the pooled positivity rates were 21.1% for CEA, 27.8% for CA19-9 (7), and 45% for preoperative CEA/CA199 (6). In clinical settings, immunological and nutritional statuses serve as practical and valuable biomarkers for GC (9), offering potential improvements in the predictive efficiency of CEA/CA199, such as NLR (10, 11), LMR (12), PLR (13, 14), SII (15), and ALB (16). However, a comprehensive evaluation of these markers’ prognostic and dynamic monitoring values remains challenging.

In this multicentre, longitudinal, retrospective cohort study, we comprehensively evaluated the predictive value of tumour biomarkers as well as immunological and nutritional indicators. Finally, we identified CEA, HGB, CA125, ALB, CA199, and NLR as the six most important prognostic indicators for patients with GC after surgery. The development of the HI-GC risk score based on these six variables allowed good prognostic accuracy and risk prediction of death, with the highest AUROC (0.767; 95% CI: 0.743, 0.791) 1 year after gastrectomy. Furthermore, according to the change in risk at different time points, we also established short- and long-term state chain models, and their Harrell’s C-indices for OS were 0.603(95%: 0.583, 0.623), 0.640 (95%: 0.622, 0.658), respectively. To the best of our knowledge, the HI-GC risk score is the first dynamic score model based on blood chemistry tests that offer real-time insights into the effectiveness of adjuvant chemotherapy in gastric cancer patients.

In the current study, we found a progressive improvement in the accuracy of the HI-GC risk score over time after surgery. Notably, the HI-GC risk score demonstrated superior predictive performance in patients who completed adjuvant chemotherapy (AUROC, 0.767; after 1 year) while showing a relative deficiency within 1 year. Additionally, in the subgroup analysis, the top two highest accuracy subgroups of risk score were patients with GC in TNM stage I (AUROC: 0.824; 95% CI: 0.560, 1.000) and aged <50 (AUROC: 0.792; 95% CI: 0.746, 0.837) after 12 months. Although the 95% confidence interval of the AUROC for gastric cancer patients in TNM stage I was wide owing to the small sample size, it still suggests the significant potential for postoperative monitoring in this subgroup of patients with TNM stage I and age <50.

Our study utilised the time-series data of the HI-GC risk score to develop short- and long-term state chain models. This novel and user-friendly method offers a simplified approach for clinicians who can simply observe the number of predictive factors at different time points to stratify patients into risk subgroups and assess their state transitions without complex calculations. Additionally, it serves as a valuable decision-making assistance system. Importantly, short-term state-chain models strongly support the recommendation of adjuvant chemotherapy for patients in the low-high and high-high risk groups. Conversely, patients in the low-low and high-low risk groups require further evaluation to identify potential beneficiaries. Furthermore, long-term state chain models suggest the potential of these models to guide differentiated follow-up strategies after 1 year.

This study has some limitations that should be considered when interpreting the results. First, our study relied on retrospectively collected serological tests from three cancer centres, which may limit the generalizability of our findings to patients with different genetic and geographic backgrounds. Second, the sample size of patients with TNM stage I GC was relatively small, which may have affected the robustness of our models. Therefore, further external validation using a prospective large-scale population is necessary to confirm the applicability of our models to a broader patient population. Third, the timing and frequency of blood tests varied among the patients, which could introduce bias, particularly in patients who underwent more frequent tests because of early death. Fourth, the availability of baseline data of patients in the external validation sets limited the validation of the HI-GC risk score. Further studies and data collection are required to validate and refine these models. Finally, while our serological model provides a convenient tool for risk stratification in clinical settings, its development is based on several common haematological indicators. In the future, we will consider additional clinical and pathological factors in conjunction with our model to develop a dynamic machine-learning model encompassing a comprehensive risk assessment.

In summary, our study developed and validated the HI-GC risk score, a practical tool for dynamically estimating the risk of death or recurrence in post-surgical patients using serological parameters. Because blood chemistry tests are widely available, the HI-GC risk score has the potential to assist frontline clinicians in optimising adjuvant chemotherapy and follow-up strategies.

## Data availability statement

The raw data supporting the conclusions of this article will be made available by the authors, without undue reservation.

## Ethics statement

The studies involving humans were approved by the ethics committee of the Xijing Hospital (Approval No. KY20192088-F-1). The studies were conducted in accordance with the local legislation and institutional requirements. Written informed consent for participation was not required from the participants or the participants’ legal guardians/next of kin because due to the retrospective design of this study, informed consent was not required.

## Author contributions

LL: Conceptualization, Validation, Writing – original draft, Writing – review & editing, Data curation, Formal analysis. WF: Data curation, Writing – original draft, Writing – review & editing. JY: Data curation, Writing – original draft, Writing – review & editing. XG: Writing – original draft, Writing – review & editing. XW: Writing – original draft, Writing – review & editing. JYY: Writing – original draft, Writing – review & editing. WH: Writing – original draft, Writing – review & editing. HX: Writing – original draft, Writing – review & editing. LY: Writing – original draft, Writing – review & editing. JJY: Writing – original draft, Writing – review & editing. JZ: Writing – original draft, Writing – review & editing. LH: Writing – original draft, Writing – review & editing. JL: Writing – original draft, Writing – review & editing. ML: Writing – original draft, Writing – review & editing. LS: Writing – original draft, Writing – review & editing. KW: Funding acquisition, Writing – original draft, Writing – review & editing. GJ: Funding acquisition, Writing – original draft, Writing – review & editing. YN: Conceptualization, Funding acquisition, Validation, Visualization, Writing – original draft, Writing – review & editing.
