# Supplementary material for: Development and validation of serological dynamic risk score to predict outcome in gastric cancer with adjuvant chemotherapy: a multicentre, longitudinal, cohort study
Source: Front Oncol. 2024 Feb 20;14:1327691. doi: 10.3389/fonc.2024.1327691 (PMC10912618; doi:10.3389/fonc.2024.1327691)
Supplement: Supplementary file 1 [file DataSheet_1.docx]

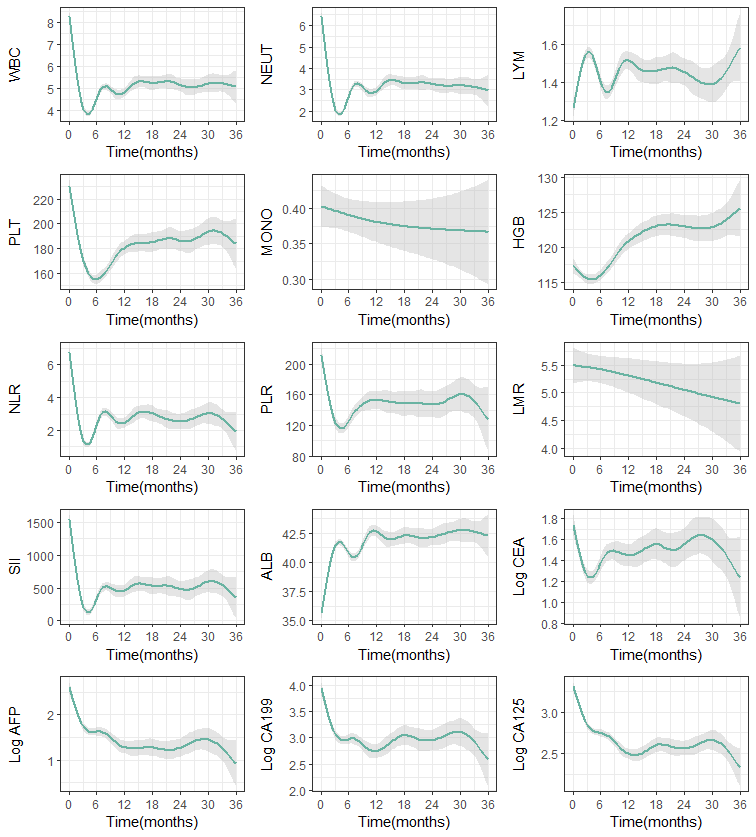


**Figure S1.** **Dynamic trajectories of 15 hematological parameters in patients with gastric cancer after surgery in the training cohort.** Smooth trajectories of the values of hematological parameters with 95% confidence intervals were plotted based on generalized additive model.


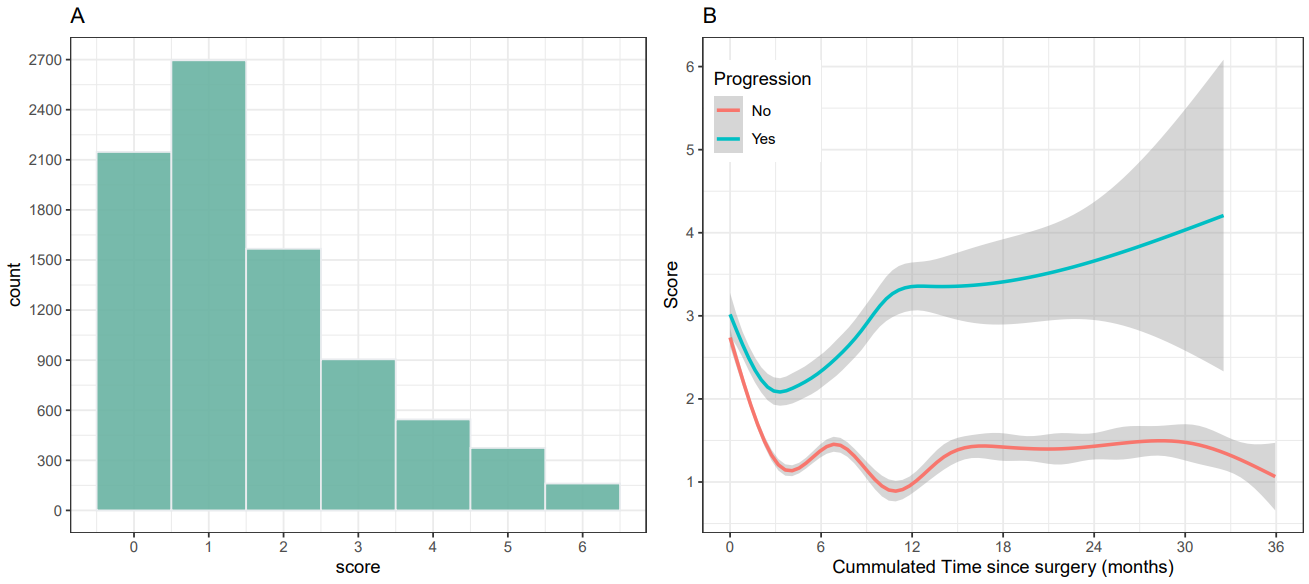


**Figure S2. (A)The chart of frequency distribution for HI-GC score.** The 80th quantile of score at 2.（B）Trajectories of HI-GC score stratified by 1-year DFS.


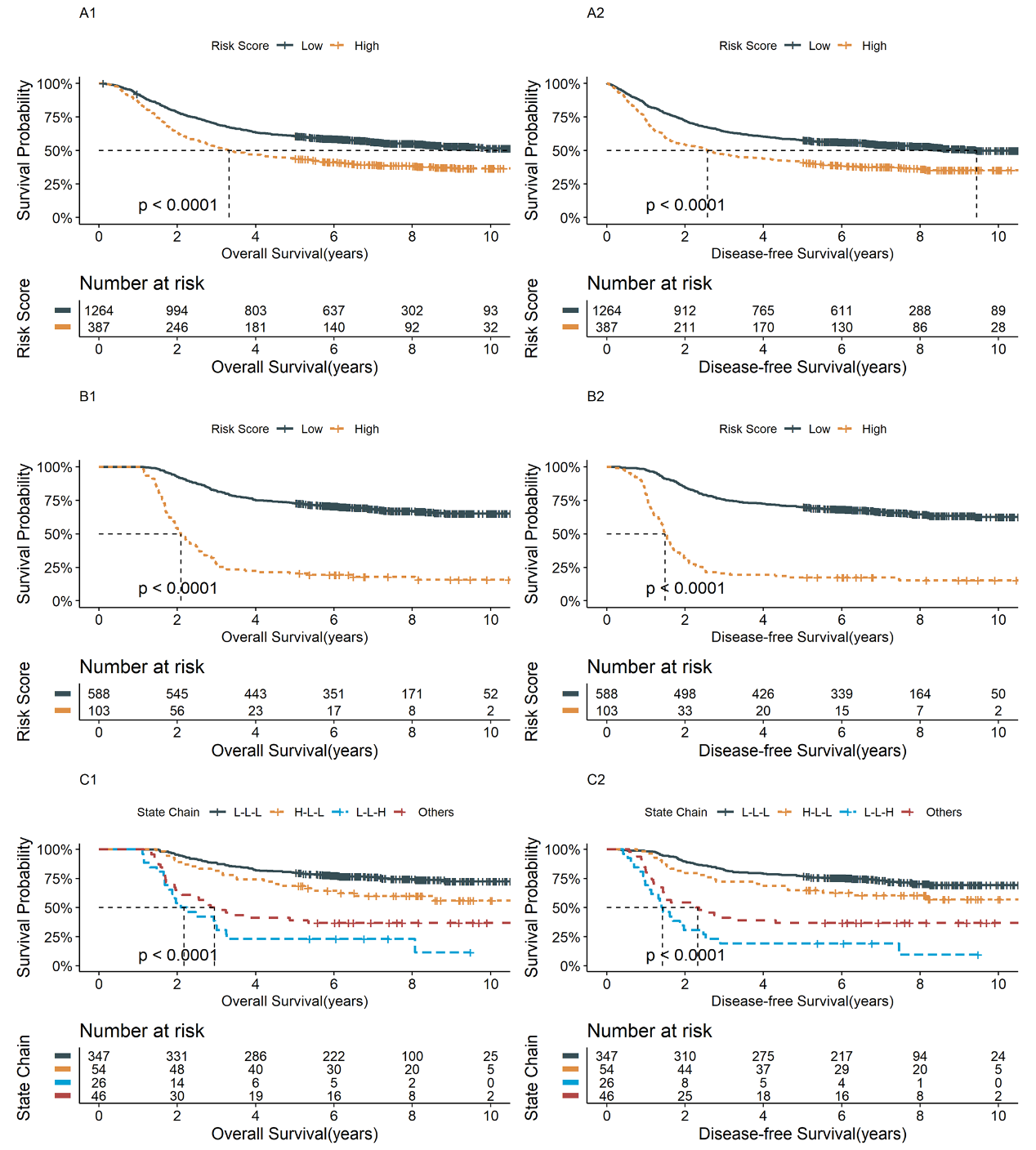


**Figure S3. Kaplan-Meier survival curves of overall survival and disease-free survival according to HI-GC score at baseline and 12 months after surgery.**


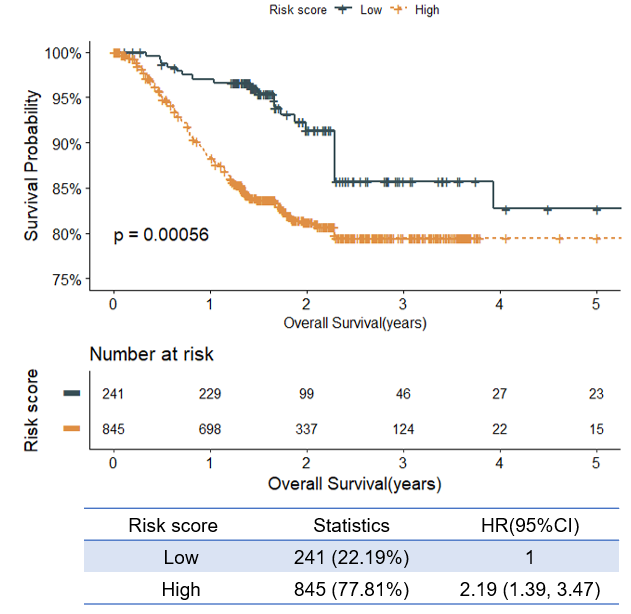


**Figure S4. Kaplan-Meier survival curves of overall survival according to HI-GC score at baseline in the validation cohort.**

**Table S1.** **Performance of NLR, PLR, LMR, and SII to predict disease progress at baseline.**

| Variable | Optimal Threshold | AUC | Accuracy | Sensitivity | Specificity | PPV | NPV |
| --- | --- | --- | --- | --- | --- | --- | --- |
| NLR | 2.5 | 0.565 | 0.556 | 0.536 | 0.576 | 0.551 | 0.560 |
| PLR | 140.8 | 0.546 | 0.547 | 0.560 | 0.534 | 0.539 | 0.555 |
| SII | 590.4 | 0.561 | 0.554 | 0.590 | 0.519 | 0.544 | 0.565 |
| LMR | 4.0 | 0.554 | 0.459 | 0.389 | 0.526 | 0.444 | 0.470 |

NLR: neutrophil/lymphocyte ratio. PLR: platelet/lymphocyte ratio; LMR: lymphocyte/monocyte ratio; SII: systemic immune inflammation index. All the optimal threshold values were determined by the Youden index through the receiver operating characteristic curve.

**Table S2. Performance of HI-GC score at different intervals from gastrectomy.**

|  | Q1 | Q2 | Q3 | Q4 |
| --- | --- | --- | --- | --- |
| Duration from  surgery(month) | 0-1 | 1-6 | 6-12 | >12 |
| No. observation(death) | 1121 | 1576 | 724 | 633 |
| No. observation(alive) | 1124 | 1553 | 853 | 811 |
| AUROC | 0.606 | 0.624 | 0.639 | 0.767 |
| 95% CI lower | 0.583 | 0.605 | 0.612 | 0.743 |
| 95% CI upper | 0.629 | 0.643 | 0.665 | 0.791 |
| Total accuracy | 0.592 | 0.590 | 0.613 | 0.736 |
| Sensitivity | 0.617 | 0.488 | 0.488 | 0.621 |
| Specificity | 0.567 | 0.694 | 0.719 | 0.825 |
| PPV | 0.587 | 0.618 | 0.595 | 0.735 |
| NPV | 0.598 | 0.572 | 0.623 | 0.736 |

**Table S3. The performance of risk score in the subgroup patients of age, gender, TNM stage, and Primary tumor location.**

|  | Q1 | | Q4 | |
| --- | --- | --- | --- | --- |
|  | Age<50 | Age ≥50 | Age<50 | Age ≥50 |
| AUROC (95% CI) | 0.583(0.536, 0.629) | 0.612(0.586, 0.638) | 0.792(0.746, 0.837) | 0.758(0.729, 0.786) |
| Total accuracy | 0.581 | 0.561 | 0.872 | 0.810 |
| Sensitivity | 0.578 | 0.629 | 0.667 | 0.603 |
| Specificity | 0.580 | 0.596 | 0.775 | 0.722 |
| PPV | 0.529 | 0.604 | 0.825 | 0.702 |
| NPV | 0.628 | 0.586 | 0.744 | 0.733 |
|  | Q1 | | Q4 | |
|  | Male | Female | Male | Female |
| AUROC (95% CI) | 0.612(0.587,0.638) | 0.583(0.534, 0.632) | 0.776(0.748, 0.804) | 0.729(0.678, 0.780) |
| Total accuracy | 0.569 | 0.558 | 0.843 | 0.761 |
| Sensitivity | 0.627 | 0.587 | 0.623 | 0.615 |
| Specificity | 0.597 | 0.573 | 0.751 | 0.689 |
| PPV | 0.583 | 0.601 | 0.741 | 0.718 |
| NPV | 0.613 | 0.544 | 0.756 | 0.667 |

|  | Q1 | | | Q4 | | |
| --- | --- | --- | --- | --- | --- | --- |
|  | TNM Ⅰ | TNM Ⅱ | TNM Ⅲ | TNM Ⅰ | TNM Ⅱ | TNM Ⅲ |
| AUROC  (95% CI) | 0.658  (0.537, 0.780) | 0.554  (0.505, 0.602) | 0.588  (0.559,0.618) | 0.824  (0.560, 1.000) | 0.658  (0.600, 0.716) | 0.761  (0.730, 0.793) |
| Total accuracy | 0.679 | 0.598 | 0.506 | 0.915 | 0.826 | 0.785 |
| Sensitivity | 0.579 | 0.506 | 0.638 | 0.750 | 0.429 | 0.665 |
| Specificity | 0.669 | 0.572 | 0.591 | 0.910 | 0.732 | 0.709 |
| PPV | 0.177 | 0.325 | 0.698 | 0.214 | 0.436 | 0.839 |
| NPV | 0.931 | 0.760 | 0.438 | 0.992 | 0.822 | 0.581 |
|  | Q1 | | | Q4 | | |
|  | Proximal | Body | Antrum | Proximal | Body | Antrum |
| AUROC  (95% CI) | 0.630  (0.591, 0.670) | 0.572  (0.528, 0.616) | 0.612  (0.576, 0.649) | 0.743  (0.695, 0.791) | 0.753  (0.707, 0.798) | 0.795  (0.759, 0.830) |
| Total accuracy | 0.550 | 0.578 | 0.573 | 0.815 | 0.798 | 0.855 |
| Sensitivity | 0.674 | 0.576 | 0.601 | 0.601 | 0.616 | 0.636 |
| Specificity | 0.612 | 0.577 | 0.586 | 0.732 | 0.718 | 0.751 |
| PPV | 0.600 | 0.581 | 0.579 | 0.673 | 0.705 | 0.799 |
| NPV | 0.627 | 0.573 | 0.594 | 0.763 | 0.726 | 0.722 |

Q1: 0-1 month. Q4: >12 month.

Table S4. Baseline characteristics of the validation cohort grouped by HI-GC risk score

| Risk score | Low(n=241) | High(n=845) | P-value |
| --- | --- | --- | --- |
| Age | 56.5 ± 11.3 | 61.0 ± 11.6 | <0.001 |
| BMI | 22.6 ± 0.6 | 22.3 ± 0.6 | <0.001 |
| Sex |  |  | 0.025 |
| Male | 197 (81.7%) | 632 (74.8%) |  |
| Female | 44 (18.3%) | 213 (25.2%) |  |
| Type of gastrectomy |  |  | 0.490 |
| Proximal | 221 (91.7%) | 759 (89.8%) |  |
| Distal | 9 (3.7%) | 48 (5.7%) |  |
| Total | 11 (4.6%) | 38 (4.5%) |  |
| Differentiation |  |  | 0.044 |
| Well | 1 (0.4%) | 25 (3.0%) |  |
| Moderate | 89 (36.9%) | 275 (32.5%) |  |
| Poor/Undifferentiation | 151 (62.7%) | 545 (64.5%) |  |
| Primary tumor location |  |  | 0.312 |
| Proximal | 54 (22.4%) | 211 (25.0%) |  |
| Body | 119 (49.4%) | 435 (51.5%) |  |
| Antrum | 68 (28.2%) | 199 (23.6%) |  |
| MVI |  |  | 0.494 |
| No | 194 (80.5%) | 663 (78.5%) |  |
| Yes | 47 (19.5%) | 182 (21.5%) |  |
| Nerve invasion |  |  | 0.593 |
| No | 216 (89.6%) | 767 (90.8%) |  |
| Yes | 25 (10.4%) | 78 (9.2%) |  |
| pN stage |  |  | 0.004 |
| 0 | 75 (31.1%) | 217 (25.7%) |  |
| 1 | 54 (22.4%) | 139 (16.4%) |  |
| 2 | 49 (20.3%) | 167 (19.8%) |  |
| 3 | 63 (26.1%) | 322 (38.1%) |  |
| pT stage |  |  | 0.004 |
| 1 | 38 (15.8%) | 91 (10.8%) |  |
| 2 | 31 (12.9%) | 63 (7.5%) |  |
| 3 | 7 (2.9%) | 21 (2.5%) |  |
| 4 | 165 (68.5%) | 670 (79.3%) |  |
| pTNM stage |  |  | 0.003 |
| Ⅰ | 50 (20.7%) | 111 (13.1%) |  |
| Ⅱ | 51 (21.2%) | 150 (17.8%) |  |
| Ⅲ | 140 (58.1%) | 584 (69.1%) |  |
| Hospital |  |  | 0.399 |
| 900th hospital | 185 (76.8%) | 626 (74.1%) |  |
| Fujian People's Hospital | 56 (23.2%) | 219 (25.9%) |  |
